# Cesarean and VBAC rates among immigrant vs. native-born women: a retrospective observational study from Taiwan Cesarean delivery and VBAC among immigrant women in Taiwan

**DOI:** 10.1186/1471-2458-10-548

**Published:** 2010-09-10

**Authors:** Jung-Chung Fu, Sudha Xirasagar, Jihong Liu, Janice C Probst

**Affiliations:** 1 Kaohsiung Municipal United Hospital, Department of Obstetrics & Gynecology. Kaohsiung, ROC, Taiwan; 2 University of South Carolina, Arnold School of Public Health, Department of Health Services Policy and Management, Columbia, SC, USA; 3 University of South Carolina, Arnold School of Public Health, Department of Epidemiology and Biostatistics, Columbia, SC, USA

## Abstract

**Background:**

Cultural and ethnic roots impact women's fertility and delivery preferences This study investigated whether the likelihood of cesarean delivery, primary cesarean, and vaginal delivery after cesarean (VBAC) varies by maternal national origin.

**Methods:**

We conducted a nation-wide, population-based, observational study using secondary data from Taiwan. De-identified data were obtained on all 392,246 singleton live births (≥500 g; ≥20 weeks) born to native-born Taiwanese, Vietnamese and mainland Chinese-born mothers between January 1 2006 and December 31 2007 from Taiwan's nation-wide birth certificate data. Our analytic samples consisted of the following: for overall cesarean likelihood 392,246 births, primary cesarean 336,766 (excluding repeat cesarean and VBAC), and VBAC 55,480 births (excluding primary cesarean and vaginal births without previous cesarean). Our main outcome measures were the odds of cesarean delivery, primary cesarean delivery and VBAC for Vietnamese and Chinese immigrant mothers relative to Taiwanese mothers, using multiple regression analyses to adjust for maternal and neonatal characteristics, paternal age, institutional setting, and major obstetric complications.

**Results:**

Unadjusted overall cesarean, primary cesarean, and VBAC rates were 33.9%, 23.0% and 4.0% for Taiwanese, 27.6%, 20.1% and 5.0% for mainland Chinese, and 19.3%, 13.9 and 6.1% for Vietnamese respectively. Adjusted for confounders, Vietnamese mothers were less likely than native-born Taiwanese to have overall and primary cesarean delivery (OR = 0.59 and 0.58 respectively), followed by Chinese mothers (both ORs = 0.90 relative to native-born Taiwanese). Vietnamese mothers were most likely to have successful VBAC (OR = 1.58), followed by Chinese mothers (OR = 1.25).

**Conclusion:**

Immigrant Vietnamese and Chinese mothers have lower odds of cesarean and higher VBAC odds than native-born Taiwanese, consistent with lower cesarean rates prevailing in their home countries (Vietnam 10.1%; mainland China 20% - 50% rural and urban respectively).

## Background

With increasing prosperity and higher educational attainment of women, Taiwan is experiencing major social changes in attitudes to marriage and child bearing. Many Taiwanese women decline or delay marriage and child-bearing, which is reflected in a rapid fertility decline. Taiwan's current total fertility rate is 1.07, among the lowest in the world [1,2]. Age at first marriage increased from 30.7 to 31.1 years for men and from 26.9 to 28.4 for women during 2004 to 2008 [3,4]. Maternal age at first delivery increased from 26.4 years in 1998 to 28.9 years in 2008[5]. Changing marital and childbearing preferences of native-born Taiwanese women has resulted in many Taiwanese men, particularly of lower socioeconomic status (SES), education, income and rural residence seeking brides from neighboring Asian countries [6,7], the vast majority being mainland Chinese and Vietnamese (12.5% of all marriages and 9.5% of births in 2008) [5,8].

Concurrent with the fertility decline among native-born Taiwanese, cesarean rates have escalated to about a third of all births, one of the highest in the world [9,10]. Cesarean delivery is associated with higher maternal and fetal risks, such as maternal mortality, re-hospitalization for wound complications and infection, placental abnormalities in subsequent births (placenta accrete, percreta and previa), uterine rupture, preterm births, neonatal complications and higher costs [11-15]. Cesarean rates by maternal ethnicity of origin have not been systematically studied.

Cultural-ethnic-national origin of women plausibly impacts fertility and birthing preferences. High cesarean rates in Latin America [16], and a low rate of 10.1% in Vietnam [17] are documented. Literature suggests that cesarean rates among immigrant women mirror the rates prevailing in their home countries. Vietnamese immigrant mothers have lower cesarean rates than the host country rate in many countries. In Switzerland, Vietnamese-immigrant mothers lower have cesarean rates than Latin American immigrants [18], and lower than the nation-wide CS rate. In Norway, Vietnamese immigrants had a 10.1% cesarean rate compared to 24.3% for Latin American immigrants from Chile and Brazil [19]. The latter rate is closer to the domestic cesarean rates in Brazil 36% (16), and Chile 40% in 1997 [20], and to the overall South American region's rate of 33% reported by the World Health Organization [21].

Apart from the cesarean rates in Vietnam, the rates in mainland China are germane to Taiwan because of the large contribution of China to Taiwan's immigrant population. China has regions with high cesarean rates [22], about 40% in urban areas [23], the rate increasing with urbanization level [24]. Cesarean rates also vary widely by region, with a four-fold difference between the highest and lowest rates [25]. In Taiwan, immigrant mothers tend to be disproportionately rural (37% vs. 27% for Taiwanese-born women) [26]. In addition, lower cesarean rates in their country of origin may predispose immigrants to lower cesarean rates. In contrast, newborns of immigrant women tend to have older fathers [27] which in turn increases the cesarean propensity [27,28].

Apart from medical indications, extraneous factors are documented in many countries. These include maternal request, insurance coverage, institutional setting, and physician characteristics [29-31]. Maternal age particularly over 35 years contributes to increased maternal request cesarean [32]. Recent trends towards respecting patient rights and autonomy in clinical decision-making have also contributed to increasing request cesareans [33]. Isolated hospital-based surveys of providers/medical record reviews suggest that maternal request was the reason for 24.9% of elective cesarean deliveries in the UK, 9% in Italy, and 7.6% in Norway [33-35]. In urban areas of southeastern China, a 60% cesarean rate is documented, half of them due to maternal request (also based on medical record review) [23].

Vaginal delivery after a cesarean birth (VBAC) is a little documented issue in Taiwan. While provider preferences remain important factors in successful VBAC, differences in VBAC rates among immigrant mothers relative to native-born mothers may partly reflect differences in maternal cultural preferences regarding cesarean delivery. Differences in successful VBAC rates by ethnicity are noted in the US, with a lower success rate among blacks [36]. Recent studies have added to the dilemma about VBAC. Conventional obstetric wisdom favored a trial of VBAC under optimal clinical conditions, until a recent report demonstrated a doubling of the risk of major maternal and fetal complications (uterine rupture, hysterectomy, increased perinatal morbidity and mortality) in trials of vaginal delivery following a previous cesarean [37]. In the US this report caused a dramatic decline in VBAC from 28.3% in 1996 to 9.1% in 2004 and 7.9% in 2005 [38,39]. Uterine rupture, a catastrophic complication is documented in about 1% (or less) among VBAC cases [40].

Internationally, there is no documentation of population-based research on primary cesarean and VBAC by ethnicity, adjusted for maternal conditions and obstetric complications. Specifically, there is no research on cesarean and VBAC likelihood among native-born relative to immigrant women in Asian countries. This study makes a unique contribution to the literature by examining population-based data from Taiwan to assess the relative likelihood of cesarean delivery (primary and overall) and VBAC among immigrant and native-born mothers. It offers clinical complication-adjusted rates for comparisons with cesarean rates in their home countries.

## Methods

The study used nationwide birth certificate data from January 2006 to December 2007 obtained from Taiwan's Bureau of Health Promotion, Department of Health, which gathers data from all institutions in Taiwan through online submissions. All singleton live births ≥ 20 gestational weeks to mothers of Taiwanese, Vietnamese and mainland Chinese origin were included, 99% of all births in Taiwan. (Other ethnicities, Indonesian, Cambodian etc were excluded due to inadequate numbers.) The study was approved by the Kaohsiung Municipal United Hospital Institutional Review Board and the University of South Carolina Institutional Review Board.

The key dependent variables of interest are primary cesarean delivery (mother's first cesarean delivery regardless of previous parity), overall cesarean delivery (across all deliveries), and VBAC (vaginal birth after cesarean), all dichotomous. The key independent variable of interest is maternal ethnicity by country of birth, Taiwanese, mainland Chinese and Vietnamese. We adjusted for maternal demographic variables (age: < 20, 20-34, and > 34 years), urbanization level (urban, suburban, rural), marital status (single, currently married), neonatal characteristics (gender, birth weight, gestational age), paternal age (≤24, 25-35, and >35 years), and medical institution setting (clinic vs. hospital). Birth weight was classified as < 1 standard deviation from the mean, between +1 and -1 standard deviation, and > 1 standard deviation). Gestational age was categorized as preterm (<37 weeks at delivery), prolonged pregnancy (≥ 42 weeks), and term pregnancy (37-41 completed weeks) [39] Per accepted practice gestational age in Taiwan is calculated based on the last menstrual period combined with sonography in the first trimester, with the latter superseding LMP when discrepant. Some senior obstetricians do not use sonography to validate LMP calculations. History of previous cesarean was controlled for assessing the overall cesarean risk.

We controlled for institutional setting because a previous study reported higher cesarean likelihood for obstetric clinics relative to hospitals in Taiwan [10]. Institutional characteristics of teaching status and ownership could not be accounted for due to lack of these variables in the birth certificate dataset. Presence of any major obstetric complication that is a reasonable clinical justification for cesarean was controlled for (one or more of the following: breech or malpresentation, dystocia, fetal distress, cephalo-pelvic disproportion, placenta previa and abruptio placentae [41]. Pregnancy complications included hypertensive disorder or diabetes. Hypertensive disorder indicates that at least two readings six hours apart showed blood pressure readings of >140 systolic and/or >90 mm diastolic, regardless of pre-existing or pregnancy-related hypertension including preeclampsia (mild or severe). Diabetic disorder in birth certificate data includes preexisting and gestational diabetes. Maternal parity was not available in the dataset.

Multiple logistic regression analyses were used to determine the adjusted odds of these delivery types for immigrant groups relative to Taiwanese. The SPSS statistical package (Version 15) was used. The study tested the following hypotheses: 1. Vietnamese women have the lowest adjusted primary cesarean and overall cesarean likelihood and the highest VBAC likelihood. 2. Taiwanese and Chinese mothers will have similar adjusted odds of primary and overall cesarean, and VBAC.

## Results

Of total 411,943 births, 392,246 births met the inclusion criteria, of which 349,730 (89.2%) were to native Taiwanese mothers, 19,866 (5.1%) to mainland Chinese, and 22,650 (5.8%) to Vietnamese mothers. Of these, 53,186 births were repeat cesarean and 2,294 VBAC births. The analytic sample for total cesarean consists of all 392,246 births, for primary cesarean 336,766 births (excluding repeat cesarean and VBAC), and for VBAC likelihood 55,480 births (after excluding primary cesarean births and vaginal births without history of previous cesarean).

Table 1 shows the distribution of births by demographic and medical risk factors. Mean maternal age was 29.3, 28.2 and 24.0 years for Taiwanese, Chinese and Vietnamese mothers. In contrast, paternal age was highest for babies of Vietnamese women (mean 37.8 years) and least for Taiwanese (32.2 years). Single status (never married, divorced, widowed or cohabitating) was more frequent among Taiwanese mothers (7.4% vs. 1% each in Chinese and Vietnamese). Rural-urban distribution shows significantly lower rural residence among Taiwanese women, 11.3% vs. 22.6% among Vietnamese, and 15.2% among Chinese). Excluding primary cesarean births, Taiwanese mothers had the highest prevalence of previous cesarean (14.9%), and Vietnamese the lowest (6.8%).

**Table 1:** Parental demographics, maternal risk factors, neonatal characteristics and institutional setting of births distributed by maternal ethnicity in Taiwan, 2006-2007*

Characteristics‡	Taiwanese, n = 349,730	Chinese, n = 19,866	Vietnamese, n = 22,650	Total, n = 392,246
	**n (%)**	**n (%)**	**n (%)**	**n (%)**

Parental characteristics				
Maternal age				
<20	9,303 (2.7)	7 (0.0)	2,080 (9.2)	11390(2.9)
20-35	298,825 (85.4)	18,491 (93.1)	20,246 (89.4)	337,562(86.1)
>35	41,602 (11.9)	1,368 (6.9)	324 (1.4)	43,294(11.0)
Paternal age (y/o)				
<25	21,893 (6.8)	166 (0.8)	211 (0.9)	22,270 (6.1)
25-35	214,519 (66.3)	7,795 (39.6)	7,601 (33.9)	229,915 (62.8)
>35	87,313 (27.0)	11,724 (59.6)	14,602 (65.1)	113,639 (31.1)
Single	26,002 (7.4%)	179 (0.9%)	236 (1.0%)	26,417 (6.7%)
History of cesarean	51,948(14.9%)	1.995(10.0%)	1,537(6.8%)	55480(14.1%)
Length of gestation				
Preterm	25,521(7.3)	839(4.2)	1287(5.7)	27,647(7.0)
Term	323,451(92.5)	18923(95.3)	21,241(93.8)	363,615(92.7)
Prolonged	758(0.2)	104(0.5)	122(0.5)	984(0.3)
Gestational age (wks),	38.4 ± 1.6	38.8 ± 1.4	38.6 ± 1.5	38.4 ± 1.6
Urbanization				
Urban	256,860 (73.4)	13,753 (69.2)	12,824 (56.6)	283,437 (72.3)
Suburban	53,324 (15.2)	3,094 (15.6)	4,699 (20.7)	61,117 (15.6)
Rural	39,546 (11.3)	3,019 (15.2)	5,127 (22.6)	47,692 (12.2)
Neonatal characteristics				
Male	182,829(52.3)	10,392(52.3)	11,806(52.1)	205,027(52.3)
Birth weight <1SD	44,280(12.7)	1,463(7.4)	2,607(11.5)	48,350(12.3)
Birth weight intermediate	259,670(74.2)	14,530(73.1)	17,408(76.9)	291,608(74.3)
Birth weight >1SD	45,780(13.1)	3,873(19.5)	2,635(11.6)	52,288(13.3)
Maternal medical complications				
Diabetes	2659(0.8)	55(0.3)	19(0.1)	2733(0.7)
Hypertension	4099(1.2)	72(0.4)	55(0.2)	4226(1.1)
Major obstetric complications	29627(8.5)	1233(6.2)	1197(5.3)	32057((8.2)
Institutional setting				
Clinic	111,776 (32.0)	7,186 (36.2)	10,290 (45.4)	129,252 (33.0)
Hospital	237,954 (68.0)	12,680 (63.8)	12,360 (54.6)	262,994 (67.0)

*Data received from the Taiwan Bureau of Health Promotion

†Differences between Taiwanese and immigrant women are significant for gestational age, P < 0.01

‡Differences between Taiwanese and immigrant women (chi-square tests) are significant for all variables (maternal age, paternal age, marriage, hypertension, diabetes, major obstetric complications, length of gestation, urbanization residence, birth weight) at a p < 0.01, except male gender with P = 0.90)

Newborn gender was similar for the three ethnicities. Babies of Chinese mothers were the most likely to weigh >1 standard deviation from the overall mean, and Taiwanese the most likely to weigh <1 standard deviation. Major obstetric complications were most prevalent among Taiwanese (8.5%), and least (5.3%) among Vietnamese. Significantly more Vietnamese (45.4%) and Chinese mothers (36.2%) gave birth in clinics, than Taiwanese (32.0%).

Table 2 presents the maternal ethnicity among overall cesarean, primary cesarean, and VBAC samples. The unadjusted overall cesarean rate was highest among Taiwanese (33.9%), followed by Chinese (27.6%), and Vietnamese (19.3%) (all statistically significant),. The primary cesarean rate pattern mimicked the overall cesarean rate, 23.0%, 20.1%% and 13.9%. VBAC rates showed a reverse pattern, 4.0%, 5.0% and 6.1% respectively. The nation-wide VBAC rate was 4.1% (2,294 VBAC among 55,480 births to women with a prior cesarean).

**Table 2:** Cesarean and VBAC rates among the three ethnic groups

	Total birth	Vaginal birth	Cesarean birth			VBAC§
				
			Primary@ cesarean	Repeat cesarean	Total Cesarean	
Taiwanese, n	349730	231266	68,617	49,847	118,464	2101
%	100.0%	66.1%	23.0%	10.8%	33.9%	4.0%
Chinese, n	19866	14384	3,587	1,895	5,482	100
%	100.0%	72.4%	20.1%*	7.5%*	27.6%*	5.0%*
Vietnamese, n	22650	18276	2,930	1,444	4,374	93
%	100.0%	80.7%	13.9%**,***	5.4%**,***	19.3%**,**	6.1%***
Total	392,246	263,926	75,134	53,186	128,320	2,294

%, Percentage of delivery mode are calculated as total number/total births

†Total cesarean = primary cesarean + repeat cesarean

‡ Total births = vaginal births + total cesarean

§ VBAC = vaginal birth after cesarean, VBAC rate = VBAC/(VBAC+ repeat cesarean)

@ Number of cesarean deliveries to women who have not had a previous cesarean regardless of parity

* Significant difference between Taiwanese and Chinese, ** Significant difference between Chinese and Vietnamese, *** significant difference between Taiwanese and Vietnamese

Table 3 shows the adjusted cesarean and VBAC odds adjusted for maternal demographics, neonatal characteristics, paternal age, medical institution type, pregnancy complications, obstetric complications, and birth weight. Relative to Taiwanese, Vietnamese mothers were the least likely to have cesarean delivery (OR = 0.58), followed by Chinese (OR = 0.90). Vietnamese were the most likely to have VBAC delivery (OR = 1.58, followed by Chinese (OR = 1.25).

**Table 3:** Adjusted odds (95% CI) of overall cesarean, primary cesarean and VBAC in Taiwan, 2006-2007

	Total cesarean (n = 128,320)		Primary cesarean, (n = 53,186)		VBAC (n = 2,294)	
			
	OR (95% C.I.)	p	OR (95% C.I.)	p	OR (95% C.I.)	p
Ethnicity						
Taiwanese*						
Chinese	0.90(0.86, 0.94)	0.000	0.90 (0.86, 0.94)	0.000	1.25 (1.02, 1.55)	0.033
Vietnamese	0.59(0.56, 0.61)	0.000	0.58(0.55, 0.61)	0.000	1.58 (1.26, 1.97)	0.000
Parental demographics						
Maternal age						
Aged 20-35*						
Aged <20	0.84(0.78, 0.91)	0.000	0.85 (0.79, 0.92)	0.000	1.51 (0.88, 2.50)	0.131
Aged >35	1.46(1.41, 1.52)	0.000	1.49(1.44, 1.54)	0.000	0.89 (0.78, 1.01)	0.062
Paternal age, y/o						
Aged <25*						
Aged 25-35	1.06(1.02, 1.11)	0.004	1.05((1.00, 1.10)	0.020	0.68 (0.53, 0.88)	0.003
Aged >35	0.98(0.94, 1.03)	0.429	0.97 (0.93, 1.02)	0.229	0.77 (0.60, 1.01)	0.052
Single	1.04(0.48,2.24)	0.930	0.96 (0.44, 2.08)	0.959	XX	
Maternal/Pregnancy factors						
Previous history of cesarean	108.6(103.7, 113.6)	0.000				
Length of gestation						
Term pregnancy*						
Preterm	1.37(1.31, 1.43)	0.000	1.43(1.37, 1.49)	0.000	1.37 (1.18, 1.59)	0.000
Prolonged pregnancy	1.94(1.65, 2.28)	0.000	2.00(1.70, 2.36)	0.000	4.00 (1.67, 9.58)	0.002
Urbanization						
Rural*						
Suburban	1.06(1.02, 11.0)	0.003	1.07 (1.02, 1.10)	0.002	1.06 (0.89, 1.25)	0.540
Urban	1.21(1.17, 1.25)	0.000	1.23 (1.20, 1.27)	0.000	1.04 (0.91, 1.20)	0.570
Neonatal characteristics						
Male	1.07(1.05, 1.09)	0.000	1.06 (1.05, 1.09)	0.000	0.88 (0.81, 0.96)	0.006
Intermediate birthweight*						
Birthweight <1SD	1.05(1.01, 1.08)	0.004	1.07(1.03, 1.10)	0.000	1.36 (1.19, 1.55)	0.000
Birthweight >1SD	1.77(1.72, 1.81)	0.000	1.81 (1.76, 1.86)	0.000	1.04 (0.91, 1.19)	0.572
Maternal medical complications						
DM	1.23(1.10, 1.38)	0.000	1.25 (1.12, 1.40)	0.000	0.99 (0.65, 1.49)	0.974
Hypertensive	8.0(7.42, 8.63)	0.000	8.04(7.45,8.67)	0.000	0.39 (0.24, 0.62)	0.000
Major Obstetric complication	80.7(76.5,85.2)	0.000	87.6(82.9,92.62)	0.000	0.32(0.27, 0.39)	0.000
Institutional setting						
Birth in clinic*						
Birth in Hospital	0.68(0.67, 0.70)	0.000	0.68(0.66 0.69)	0.000	2.11 (1.89, 2.35)	0.000

*Reference group

†OR = odds ratio

‡ VBAC = vaginal birth after cesarean

§Major maternal complication refers to presence of one or more of the following that reasonably justifies a cesarean delivery. It includes dystocia, cephalopelvic disproportion, breech/malpresentation, placenta previa, placental abruption, fetal distress.

XX: No single mothers in the VBAC sample, hence marital status not used in this model.

Overall cesarean, primary cesarean, and VBAC were significantly associated with medical institution type, urbanization, maternal age, paternal age, gestational period, infant birth weight, gender, obstetric and pregnancy complications, and, in the overall cesarean analysis, history of cesarean delivery. Cesarean delivery was less likely among rural residents, and in a hospital setting (relative to clinic, OR = 0.68; p = 0.000). Hospital setting was associated with higher VBAC likelihood (OR = 2.15; p = 0.000). Birth weight of <1SD and >1 SD were significantly associated with cesarean (OR = 1.05; P = 0.000, and OR = 1.77; p = 0.000 respectively relative to intermediate weight). Increased cesarean likelihood is noted with advanced maternal age (>35 years) (OR = 1.46; p = 0.000), preterm birth (OR = 1.37), prolonged pregnancy (OR = 1.94), maternal diabetes (OR = 1.23), hypertension (OR = 8.0), history of previous cesarean (OR = 108.6), and major obstetric complications (OR = 80.7)) all p = 0.000.

VBAC was associated with prolonged pregnancy relative to term pregnancy (OR = 4.0, p = 0.002), and with lower birth weight <1 SD (relative to intermediate birth weight; OR = 1.36, p = 0.000),. VBAC was significantly less likely with paternal age 25-35 years relative to younger fathers (OR = 0.68, p = 0.003), and with hypertensive disorder (OR = 0.39, p = 0.000).

To verify the findings sensitivity analyses were conducted, stratifying the total sample by the major cesarean risk factors of maternal age, paternal age, history of previous cesarean, urbanization, birth weight, and institutional setting. Table 4 presents the adjusted cesarean and VBAC odds (adjusted for the remaining variables) in each of these sub-samples stratified on risk factors. Within all risk categories except for paternal age <25 years and maternal age >35 years, Vietnamese in all sub-samples have significantly lower cesarean likelihood and the Chinese are in between, particularly marked among women <35 years, and among fathers aged >25 years.

**Table 4:** Sensitivity analysis: Adjusted odds of cesarean vs. vaginal birth for native-born Taiwanese, Chinese and Vietnamese mothers among sub-groups categorized by maternal age, paternal age, previous cesarean, urban/rural, birth weight, and institutional setting

Stratification variable	Chinese		Vietnamese		Taiwanese
	
	Unadjusted	Adjusted	Unadjusted	Adjusted	Reference
Maternal age					
Aged <20, n = 11390	0.70(0.08, 5.79)	0.84(0.10, 7.27)	**0.74(0.65, 0.84)**	**0.79(0.64, 0.98**	1
Aged 20-35, n = 337562	**0.76(0.74, 0.79)**	**0.86(0.83, 0.90)**	**0.50(0.48, 0.52)**	**0.57(0.54, 0.59)**	1
Aged >35, n = 43294	**0.73(0.66, 0.82)**	1.06(0.94, 1.21)	**0.80(0.64, 0.99**	1.23(0.96, 1.60)	1
Paternal age (y/o)					
<25, n = 22270	0.93(0.65, 1.33)	0.97(0.66, 1.43)	0.73(0.52, 1.03)	0.79(0.54, 1.14)	1
25-35, n = 229915	**0.74(0.70, 0.78)**	**0.86(0.81, 0.91)**	**0.47(0.44, 0.50)**	**0.57(0.53, 0.61)**	1
>35, n = 113639	**0.60(0.57, 0.62)**	**0.89(0.84, 0.94)**	**0.37(0.35, 0.38)**	**0.59(0.56, 0.62)**	1
History of previous cesarean					
Yes, n = 55480	**0.80(0.65, 0.98)**	**0.78(0.62, 0.96)**	**0.65(0.53, 0.81)**	**0.61(0.49, 0.77)**	1
No, n = 336766	**0.84(0.80, 0.87)**	**0.89(0.85, 0.92)**	**0.54(0.52, 0.56)**	**0.59(0.57, 0.62)**	1
Urbanization					
Urban, n = 285771	**0.74(0.72, 0.77)**	**0.88(0.85, 0.92)**	**0.47(0.45, 0.49)**	**0.57(0.56, 0.62)**	1
Suburban, n = 61117	**0.78(0.72, 0.85)**	**0.90(0.81, 0.99)**	**0.49(0.45, 0.53)**	**0.61(0.56, 0.67)**	1
Rural, n = 45359	**0.72(0.65, 0.79)**	**0.80(0.71, 0.90)**	**0.46(0.43, 0.50)**	**0.59(0.53, 0.65)**	1
Birthweight					
Birthweight <1SD, n = 48350	**0.76(0.68, 0.85)**	0.91(0.80, 1.03	**0.50(0.46, 0.55)**	**0.69(0.62, 0.77)**	1
Birthweight ± 1SD, n = 291608	**074(0.71, 0.77)**	**0.86(0.82, 0.90)**	**0.46(0.44, 0.48)**	**0.57(0.54, 0.60)**	1
Birthweight > 1SD, n = 52288	**0.69(0.64, 0.74)**	**0.86(0.79, 0.93)**	**0.50(0.45, 0.54)**	**0.66(0.59, 0.73)**	1
Institutional setting					
Clinic, n = 129252	**0.74(0.70, 0.78)**	**0.84(0.79, 0.90)**	**0.44(0.42, 0.47)**	**0.58(0.54, 0.62)**	1
Hospital, n = 262994	**0.69(0.64, 0.74)**	**0.86(0.79, 0.93)**	**0.50(0.46, 0.54)**	**0.66(0.59, 0.73)**	1

* Odds ratios with statistical significance are in bold type. Adjusted odds are obtained after adjusting for all variables other than the categorization variable, per the list of variables used for the full sample, Table 3.

## Discussion

The pattern of findings, particularly the consistency of the ethnic associations within risk-stratified sub-groups support the hypothesis that cesarean delivery type, both primary and repeat cesarean may be influenced by maternal attitudes, which are likely shaped by the prevailing cultural attitudes in women's country of origin. Taiwan's Vietnamese immigrants show almost identical rates to immigrant Vietnamese in Norway [19] and to their home country [17]. Another validating finding in our study is that VBAC rates, which depend on maternal acceptability of a trial of labor follows the pattern observed with cesarean rates, although the magnitude of VBAC rates itself is low. Collectively, therefore the evidence suggests maternal cultural mindset as a possible factor driving these consistent inter-ethnic differences.

To our knowledge, this is the first comprehensive study to test this hypothesis using population-based data, accounting for most of the influential demographic and medical risk factors, and also validating the findings among risk-stratified sub-groups. Vietnamese immigrants' low primary cesarean rate that is similar to their home country rate of 10.1% [17] may reflect a culturally-rooted lower preference for cesarean even after adjustment for demographic and medical risks. Chinese immigrants have lower CS rates than native Taiwanese, and lower rates than highly urbanized parts of China [23]. The latter could be due to immigrants hailing from rural or less-urbanized parts of China.

Taiwan presented a unique natural experiment and healthcare environment to test this hypothesis. It has nation-wide health insurance coverage with a comprehensive, uniform health benefit plan, very low co-payments, and importantly, free universal coverage of maternal and neonatal care regardless of citizenship status. Second, since 2005 the Bureau of National Health Insurance reimburses deliveries at the same price for both vaginal delivery and cesarean, with a 10% premium for VBAC, reducing the financial incentive for cesarean that prevails in many countries. Cesarean delivery is no doubt also partly driven by physician preferences, particularly the predictable cost and timing of a cesarean delivery relative to the uncertain timing and round-the-clock readiness and resources necessitated by waiting for spontaneous vaginal delivery. However, physicians' preferences regarding delivery type are unlikely to vary systematically by patient ethnicity, although no documented studies from Taiwan are available on this topic.

Third, because of a single payer system, uniform coding and risk documentation procedures are used, which reduces the likelihood of non-random bias in co-morbidity and risk factor documentation. Fourth, Taiwan has a significantly high volume of recently initiated social immigration (immigration by marriage), with immigrants plausibly retaining their native cultural mindset. Being an island nation, Taiwan has successfully contained immigration volumes to legal immigration, most of it within the last two decades, thus presenting a homogenous, recent immigrant population. Finally, the immigrants are of East Asian descent, though ethnically and culturally diverse. The combination of these conditions provides a unique window of opportunity to study true differences in cesarean propensity and preferences, without confounding by care access or differential acculturation into the destination country's culture.

Past international studies of immigrants vs. native-born populations have some limitations because of the use of minimally descriptive datasets when national or regional population-based datasets involving birth certificate data are used [42]. Birth certificate data lack information on urbanization and healthcare coverage type, both important correlates of delivery choices by patients and physicians [42]. The latter specifically confounds the findings due to differential financial risk borne by patients which in turn would be expected to influence both prenatal care and selection of delivery type by providers and patients. Our study, based in a single-payer, universal access system minimizes this source of confounding. Studies based on insurance claims data or chart reviews also lack information on key variables such as the plan benefit structure, payer type (insurance or self-pay), and affordability. Many birth certificate datasets also lack the variables of maternal and obstetric complications [43]. Finally studies with adequate clinical and demographic controls tend to be have used data from a single (or few) institutions [44] with potential selection bias. Additionally, our study makes a major contribution to the literature in differentiating between primary and secondary cesarean and in controlling for neonatal characteristics.

Our finding of higher cesarean rates in urban areas is consistent with studies from China [24] and Brazil [45], attributed to higher provider density, capacity of health care system, fear of malpractice litigation in urban areas [25], and relatively lower fertility rates in urban areas [46,47]. Our findings of increased cesarean at advanced maternal age are also consistent with documented studies [32]. Our finding of increased cesarean propensity at clinics relative to hospitals is consistent with other studies in Taiwan [48]. Our finding of a lack of association with advanced paternal age contradicts an earlier study [27] which, however did not adjust for urbanization and history of previous cesarean.

Maternal preference is an important nonmedical factor, but not generally recorded in claims data because the NHI does not reimburse clinically unnecessary cesarean. The consistently high rate of breech presentation recorded in medical claims in Taiwan of 8-12% [6,49] and of malpresentation in general may represent indirect evidence of diagnostic upcoding to overcome this reimbursement issue. Taiwan's breech rate contrasts with the international literature documenting 2.7% to 3.2% [49,50]. Additionally, the observed significant proportion of cesarean cases without a documented obstetric or maternal complication may mask maternal request or physician preference cases. In our data while the total cesarean rate was 32.7%, only 23.3% had a documented obstetric or maternal complication. (Our study's 8.5% rate of all obstetric complications including breech represents the widely prevalent under-documentation of maternal complications in birth certificate data as the documentation of these variables is not statutory, nor is such documentation linked to financial reimbursement.)

The study lacks data on immigrants' duration of residence in Taiwan, which would impact immigrants' health-related behaviors and decision-making [51,52]. Our dataset also lacks information on key maternal characteristics such as SES, education, prenatal care, parity, pre-pregnant weight and height. Low SES and education are associated with lower cesarean rates internationally. To the extent that rural and less urbanized residence is associated with lower SES, our study partly controls for confounding by SES. Parity is another important missing variable, which may confound our findings.

Another limitation is low coding accuracy for maternal and obstetric complications in birth certificates, incomplete or misclassified data entry relative to medical claims data [53-55]. There are no validation reports on Taiwan's birth certificate data. Therefore variables such as dystocia and fetal distress (particularly subject o over-diagnosis or misrepresentation) could be unreliable. However, there is no reason to expect bias in underreporting, misclassification or over-diagnosis. Notably, our findings show significantly lower CS likelihood among Vietnamese, after controlling for these variables, suggesting that inter-ethnic differences in cesarean propensity are real, beyond the impact of these clinical need variables. Because our study is based on secondary data analysis, it is not possible to identify the factors driving the observed differences. While culturally-conditioned preference of mothers may be a factor, another likely factor is systematic variation in provider-mother interactions by maternal ethnicity. Currently no documented studies are available on mother-obstetrician relationships in Taiwan.

## Conclusion

Immigrant mothers from mainland China and Vietnam had lower total and primary cesarean rates than native-born Taiwanese. This is evident in both unadjusted rates and in the adjusted likelihood for clinical and demographic characteristics. Our full sample findings are supported by sensitivity analyses among sub-samples stratified within risk factors, which duplicate the full sample findings. Finally, the adjusted cesarean odds among the ethnic groups are mirrored by VBAC odds. Collectively, our findings support the hypothesis that immigrants from low cesarean countries are less likely to have cesarean delivery and more likely to have successful VBAC.

## Competing interests

The authors declare that they have no competing interests.

## Authors' contributions

JF carried out the research as part of his doctoral dissertation at the University of South Carolina under the direction of SX, Major Professor. JF also prepared the manuscript draft. SX participated in planning the research, conducting the analysis, preparing the manuscript, finalizing the manuscript and making revisions. JL and JP served as dissertation committee members and made important contributions to the research methods, presentation of the results, and manuscript organization. All authors read and approved the final manuscript.

## Pre-publication history

The pre-publication history for this paper can be accessed here:

http://www.biomedcentral.com/1471-2458/10/548/prepub
